# Assessment of differences in morphological and physiological leaf lodging characteristics between two cultivars of *Hippeastrum rutilum*

**DOI:** 10.1186/s12870-020-02784-8

**Published:** 2020-12-14

**Authors:** Zhenjie Shi, Qianjiao Zheng, Xiaoyang Sun, Fuchun Xie, Jian Zhao, Gaoyun Zhang, Wei Zhao, Zhixin Guo, Ariuka Ariunzul, Shah Fahad, Muhammad Adnan, Dong Qin, Shah Saud, Chen Yajun

**Affiliations:** 1 College of Horticulture & Landscape Architecture, Northeast Agricultural University, Harbin, 150030 China; 2 Department of Agronomy, The University of Haripur, Haripur, 22620 Pakistan; 3 Department of Agriculture, University of Swabi, Khyber Pakhtunkhwa, Pakistan

**Keywords:** Lodging, Lignin, Karyotype analysis, Chlorophyll fluorescence, *Hippeastrum rutilum*

## Abstract

**Background:**

Environmental lodging stress, which is a result of numerous factors, is characterized by uncertainty. However, several studies related to lodging in cereal crops have reported that lodging in the *Hippeastrum rutilum* environment is very rare. *Hippeastrum rutilum* is a garden flower with high ornamental value and abundant germplasm resources. Under past cultivation practices, it was found that the plant types of ‘Red Lion’, with red flowers, and ‘Apple Blossom’, with pink flowers, are quite different. The leaves of ‘Red Lion’ are upright, while the leaves of ‘Apple Blossom’ show lodging, which seriously affects its ornamental value. The aims of this study were to compare the differences between the two varieties with leaf lodging and upright leaves according to morphological and physiological attributes. In this study, karyotype analysis and phenotypic morphological and physiological characteristics were compared to explore the differences between the two plant types.

**Results:**

The karyotype analysis of the two cultivars showed that their chromosome types were both tetraploid plants. The results showed that the lignin content in the leaves of ‘Red Lion’ was high, the cross-sectional structure of the leaf vascular bundle was more stable, and the chlorophyll content was high. In addition, significantly less energy was transferred to the electron transport chain (ETR) during the photoreaction. Similarly, the results regarding the maximum photosynthetic rate (Fv/Fm), nonphotochemical quenching (NPQ) and effective quantum yield of photosystem II photochemistry (△F/Fm′) all indicated that the photosynthetic capacity of “Red Lion” was greater than that of “Apple Blossom”, which was affected by leaf lodging. The size of the leaves was significantly smaller, and the leaf sag angle, leaf width, and leaf tip angle presented significantly lower values in ‘Red Lion’ than in ‘Apple Blossom’, which exhibits leaf sag. The difference in these factors may be the reason for the different phenotypes of the two cultivars.

**Conclusion:**

The results of this study proved that lodging affects the photosynthetic capacity of *Hippeastrum rutilum* and revealed some indexes that might be related to leaf lodging, laying a theoretical foundation for cultivating and improving new varieties.

## Background

*Hippeastrum rutilum* is a generic name for all species of *Hippeastrum* herbs. *Hippeastrum rutilum* is native to tropical South America; it produces very high-quality bulbous flowers, has abundant germplasm resources and is widely used in gardens [1]. However, under previous cultivation practices, it was found that the plant types of two cultivars of the species differ greatly. ‘Red Lion’ (flower color is red) is a cultivar with upright leaves, and ‘Apple Blossom’ (flower color is pink) is a cultivar that is prone to leaf lodging. The lodging characteristics of its leaves cause the ornamental quality of *Hippeastrum rutilum* to decrease, which is not conducive to its promotion and application in the gardening industry.

Lodging is a major integrated agronomic trait in plant growth and crop production. It affects the yield, quality and mechanical harvesting efficiency of crops and vegetables and the ornamental quality of flowers and the type of plants [2, 3]. Lodging is regulated by a variety of factors, such as cultivation practices, the growth environment, nutritional conditions, exogenous material regulation, internal physiological structure, and genotypic differences [4–7]. The problem of plant lodging has been a hot research topic. Researchers using chromosome segment substitution lines of rice (*Oryza sativa* L.) identified an effective quantitative trait locus (QTL) for culm strength and provided methods that can be used to improve lodging resistance and increase yield [8]. It was found that wheat (*Triticum aestivum* L.) lodging resistance was significantly related to anatomical features such as the mechanical tissue, weight of low internodes, and width of stem walls [9]. Sunflowers (*Helianthus annuus* L.) of two genotypes with different susceptibilities to lodging are affected by crop population densities, moment of force failure and the function of the stem in plants [10].

Plant lodging traits are affected by a combination of internal and external factors. Morphologically, the lodging resistance of plants is related to fiber mechanical properties, the cell wall composition, stalk morphology, morphological and mechanical attributes of the roots, and the area, size and number of vascular bundles in the stem and other factors [11–13]. Physiologically, the lodging resistance of plants is related to factors such as the composition and content of lignin, cell wall chemical components and silicon content, related enzyme activities, stem water content, and carbohydrate accumulation [3, 14, 15]. Researchers have studied plant lodging using a variety of molecular biology methods, including the combination of GWAS analysis and transcriptome sequencing. In *Brassica napus*, genes for the regulation of lignin were identified, including glycosyl hydrolase (BnaA01g00480D) and CYT1 (BnaA04g22820D), and two genes encoding the transcription factors SHINE1 (ERF) and DAR6 (LIM). The elucidation of the genetic regulation of lignin provides new perspectives [16]. *MicroRNA528* was found to affect maize resistance by controlling lignin biosynthesis under nitrogen-rich conditions [17]. The mutation of *OsCESA9* conservative sites reduces cellulose DP and crystallinity in rice and affects lodging resistance [18]. Genome-wide association analysis (GWAS) for lodging tolerance identified markers associated with lodging tolerance [19]. Two anti-lodging QTLs were identified by using AFLP, SRAP, and SSR molecular markers [20]. The *prl5* gene in rice improves lodging resistance by delaying leaf senescence and increasing carbohydrate accumulation in the stem [21]. Many external factors have an influence on lodging, including diseases, wind, rain, topography, nitrogen fertilizers, soil types, forecrops, tillage, varieties, seed rates, and sowing dates [22]. Spraying paclobutrazol (PP333) or gibberellic acid (GA3) on winter wheat (*Triticum aestivum* L.) can change the physical strength of the basal internodes and the accumulation of lignin and related enzymes [23]. The plant growth regulator trinexapac-ethyl (TE) increases ryegrass (*Lolium perenne* L.) seed yields by delaying the onset of lodging [24]. Established pea-oat intercropping systems can effectively prevent lodging [25]. The application of an appropriate amount of nitrogen and planting density can reduce rape lodging [26]. In addition, increased plant lodging is caused by certain diseases and bad weather. For example, sheath blight reduces the stem breaking resistance and increases the lodging susceptibility of rice plants [27]. Strong wind causes summer corn to lodge before the tasseling stage, which affects the yield [28]. Lodging has a negative influence on both the yield and yield quality. Photosynthesis is an important way for plants to synthesize organic matter. Setter evaluated that lodging reduces the light interception capacity of plants in rice, and hurts canopy photosynthesis and yield [29]. Chlorophyll fluorescence can be used as a probe for photosynthesis research. The maximum quantum yield of PS II is Fv/Fm = (Fm-Fo)/Fm, which reflects the potential maximum photosynthetic capacity of plants. Chlorophyll fluorescence can also be used as a measure of the original reaction of photosynthesis, carbon assimilation, and electron transfer [30–32].

To date, studies on plant lodging have mostly focused on crops with stalks, while research on the lodging of ornamental plants is rare. Studies on the mechanism of leaf lodging in *Hippeastrum rutilum* have rarely been reported. This study compares the differences between two varieties showing leaf lodging and upright leaves from the aspects of genetics, morphology, and physiology and lays a theoretical basis for the future improvement of *Hippeastrum rutilum* varieties.

## Methods

### Material selection and treatment

#### Material selection

The experimental materials used in this study included two cultivars identified among various *Hippeastrum rutilum* genotypes. The leaves of ‘Red Lion’ red flower cultivar are erect and straight, but the leaves of the ‘Apple Blossom’ pink flower cultivar exhibit lodging and sagging, and the leaf morphology of the two cultivars is quite different. The research material consisted of three-year-old mature plants and was provided by Beijing Plante Horticulture Co., Ltd., a high-tech agricultural company operating at the Institute of Environment and Sustainable Development in Agriculture of the Chinese Academy of Agricultural Science (CAAS).

#### Material treatment

The ‘Red Lion’ and ‘Apple Blossom’ test materials were planted in 10 pots and cultured in a greenhouse at the Horticultural Experimental Station of Northeast Agricultural University. The cultivation soil was uniformly prepared from compost soil, humus soil and sand at 4:4:2, and the pH was controlled between 5.5 and 6.5. The cultivation conditions required the temperature to be controlled at 18–22 °C and good ventilation, sufficient sunshine, and a humid climate. To provide the most suitable growing environment for the experimental materials, the experimental materials were cultivated in greenhouses of the Horticultural Experimental Station of Northeast Agricultural University from cultivation to the end of experiment.

### Karyotype analysis

The apical roots of well-grown plants of the two cultivars were selected as the test materials, and the chromosomes were stained and observed by in situ fluorescence hybridization (FISH). The specific experimental process was as follows:

#### Material pretreatment

A root tip was cut 1.5 cm, transferred to a wet 0.5 ml EP tube with a perforated lid, and treated in a tube filled with 10 atm of N_2_O gas for 2 h.

#### Material fixation

After pretreatment, 90% glacial acetic acid was added to the EP tube, and fixation was performed on ice for 10 min.

#### Material dissociation

After the end of fixing treatment, the tube was washed three times with distilled water until there was no obvious glacial acetic acid smell in the tube. The apical growth point was excised, placed in an enzymatic mixture (1% pectinase and 2% cellulase in 1x citrate buffer), and enzymatic hydrolysis was performed in a water bath at 37 °C for 54 min.

#### Preparation before fluorescence in situ hybridization

The treated material was washed twice with 70% ethanol, and the root tips were cut with a dissecting needle and centrifuged at 5000 rpm for 30 s. Then, the supernatant was discarded, the centrifuge tube was inverted, and the pellet was dried. According to the amount of precipitation, 30–50 μl of glacial acetic acid was added, and contents of the tube were mixed well. Seven microliters of the cell suspension were dropped onto a glass slide to prepare a smear. Under a phase-contrast microscope, the smears with better shunt directions were screened for fluorescence in situ hybridization.

#### Fluorescence in situ hybridization

The slides used for fluorescence in situ hybridization were placed in a UV cross-linker (125 mJ/cm2) for cross-linking. The prelabeled probe was diluted with 2x SSC and 1 x TE, and the dilution factor of the probe was adjusted according to the copy number of a target sequence. All the following steps had to be performed in the dark. To identify the cells on a slide, 8 μl of the diluted sample was added and then covered with a coverslip. All slides with samples were placed in an aluminum box, and the paper in the foil box was kept moist. The aluminum box was placed in boiling water, and the cells on the glass slide were deformed and hybridized with the probe at a temperature higher than 85 °C. The humidor was placed in a 55 °C incubator for preheating. After 5 min of hybridization, the slides were quickly removed and placed in a preheated humidifier, in which they were reacted overnight at 55 °C.

#### Dyeing and detection

The reaction slide was placed in 2× SSC, and a small amount of DAPI was added in a dropwise manner. Then, the slide was covered with a cover glass and observed under a microscope. Relatively well-distributed chromosome smears in the mid-term were selected and stored in a − 80 °C ultra-low-temperature refrigerator for later use.

#### Measurement of chromosome length

Clear and well-distributed chromosome cells were selected for photomicrography, and Adobe Photo-shop 2018 was used for chromosome pairing and length measurements.

The following calculations were performed for the data:

$$ \mathrm{Arm}\ \mathrm{ratio}\ \left(\mathrm{r}\right)=\mathrm{Long}\ \mathrm{arm}\ \left(\mathrm{R}\right)/\mathrm{Short}\ \mathrm{arm}\ \left(\mathrm{L}\right) $$

$$ \mathrm{Relative}\ \mathrm{chromosome}\ \mathrm{length}\ \left(\%\right)=\mathrm{Chromosome}\ \mathrm{length}/\mathrm{Total}\ \mathrm{length}\ \mathrm{of}\ \mathrm{chromosome}\ \mathrm{group}\times 100\%\mathrm{Karyotype}\ \mathrm{asymmetry}\ \mathrm{coefficient}\ \left(\mathrm{As}.\mathrm{K}\%\right)=\mathrm{Total}\ \mathrm{length}\ \mathrm{of}\ \mathrm{long}\ \mathrm{arm}/\mathrm{Total}\ \mathrm{chromosome}\ \mathrm{length}\times 100\% $$

### Determination of leaf morphology indicators

#### Leaf morphological indicators

In this test, measurement devices (ruler and protractor) were used to calculate the both angle of the tip and the width of leaf (measuring the width of a leaf at a distance of 5 cm from the tip of that leaf) and the angle of sagging for both the ‘Red Lion’ and ‘Apple Blossom’ cultivars.

#### Leaf stoma characteristics

Two samples of the leaves of ‘Red Lion’ and ‘Apple Blossoms’ were collected and the leaf area size is 0.5 cm*0.5 cm. The upper and lower epidermis were separated with tweezers and placed on a glass slide for tableting; the measurements for each sample were repeated five times. Using a Nikon-E200MV biomicroscope, epidermal cells, stomatal length, and stomatal morphology were measured in the same replicates from the two cultivars.

#### Leaf vascular bundle cross-cut structure

The paraffin section method was adopted to evaluate the anatomical structure of *Hippeastrum rutilum* leaves. Fully developed leaves were randomly selected from the two cultivars with different phenotypes, and 0.5 cm*0.5 cm segments were cut from the middle portion of the leaves. The materials were then placed in a formalin-acetic acid-alcohol mixture (FAA) for tissue fixation. After fixation, the segments underwent a series of treatments as described previously [33]. Finally, anatomical investigations were carried out on slices (8 μm thick) of the leaves using an optical microscope (BX61, OLYMPUS, Japan), and images were obtained with a digital camera. The size of the vascular bundles and midvein catheters were also observed.

### Determination of leaf physiological indicators

#### Lignin content

Referring to the method of Syros with slight modifications, the lignin content of *Hippeastrum rutilum* leaves was determined [34]. Fresh samples (0.5 g) were weighed into a mortar and ground to a homogenate by adding 95% ethanol, and the precipitate was collected after centrifugation at 4500 rpm for 10 min. The pellet was washed 3 times with an equal volume of a 1:1 95% ethanol and n-hexane solution, after which the precipitate was collected and dried. The dried product was dissolved in 0.5 ml of 25% glacial acetic acid and then left to stand in a water bath at 70 °C for 30 min. Thereafter, 0.9 ml of 2 mol/L NaOH was added to terminate the reaction. Five milliliters of glacial acetic acid and 0.1 ml of 7.5 mol/L hydroxylamine hydrochloride were added. After mixing and centrifugation of the samples at 4500 rpm for 5 min, 0.1 ml of the supernatant was aspirated and diluted with 3.0 ml of glacial acetic acid. A microplate reader was used to test the absorbance of the solution at A280 nm, and the measurements of each sample were repeated four times.

#### Leaf relative water content

The relative water content (RWC) of *Hippeastrum rutilum* leaves was determined by the weighing method. Leaf samples were collected, and the fresh weight of the leaves was determined, after which they were soaked in distilled water for 5 h to fully saturate the samples. The surface moisture was subsequently wiped off of the leaves, and the saturated fresh leaf weight was immediately measured. Finally, the samples were placed in a drying box at 105 °C for 15 min for anti-blue treatment. The samples were dried in an oven at a constant temperature of 80 °C for 12 h, and the dry weight was measured.

#### Leaf chlorophyll content

The chlorophyll content was determined in reference to an ethanol-acetone mixture extraction method. Plant leaf samples were collected, and 0.1 g of each sample was weighed into a 5 ml EP tube. Then, 2 ml of 95% ethanol was added. The measurements of each sample were repeated 4 times. After 72 h of treatment in the dark, 320 μl of the extract was pipetted, and the absorbance was measured on a microplate reader. The absorbance was measured at 665 nm, 649 nm, and 470 nm. The whole process was carried out in a dark environment to reduce the effect of light on chlorophyll decomposition.

#### Leaf chlorophyll fluorescence

An Imagining-PAM (MAXI) system (WALZ, Germany) was used to determine the chlorophyll fluorescence parameters of *Hippeastrum rutil*e. The plants were first treated for 20 min in a dark environment after the samples were placed within the probe range of the fluorometer, and the instrument parameters were set appropriately for the measurement of *Hippeastrum rutilum*. The first step was to set the light measurement parameters so that the value (Ft) was regulated within a range of 0.1–0.2 after determining the selected sample AOI region. Then, Live Video option was selected on the image page, and LED was changed so that the infrared image sample was clear. The second step is to set on saturated pulse light parameters for plant. The frequency of saturated pulse light suitable for the plant is 20s/times and the intensity is 4000umol/m^2^/s. The third step is to set for parameters of actinic light. The optimal light intensity for optimal fluorescence kinetic curve of plant is 86umol/m^2^/s. Thereafter, we set the parameters for absorptivity according to the nameplate (red gain = 1, red intensity = 49, NIR intensity = 33). After the settings were applied, on *Hippeastrum rutilum* chlorophyll fluorescence parameters could be measured.

### Statistical analysis

We performed Wilcoxon test to examine the differences between the two cultivars of *Hippeastrum rutilum*. *p* ≤ 0.05 was considered statistically significant. All statistical tests were performed using SPSS 22.0 for Windows (SPSS, USA). All charts were produced with GraphPad Prism 7.0 (GraphPad, USA) and Word 2016 (Microsoft, USA). All images were touched up and labelled using Photoshop 2018 (Adobe, USA) and Illustrator CS4 (Adobe, USA).

## Results

### Karyotype analysis between the two cultivars of *Hippeastrum Rutilum*

**Fig. 1:**
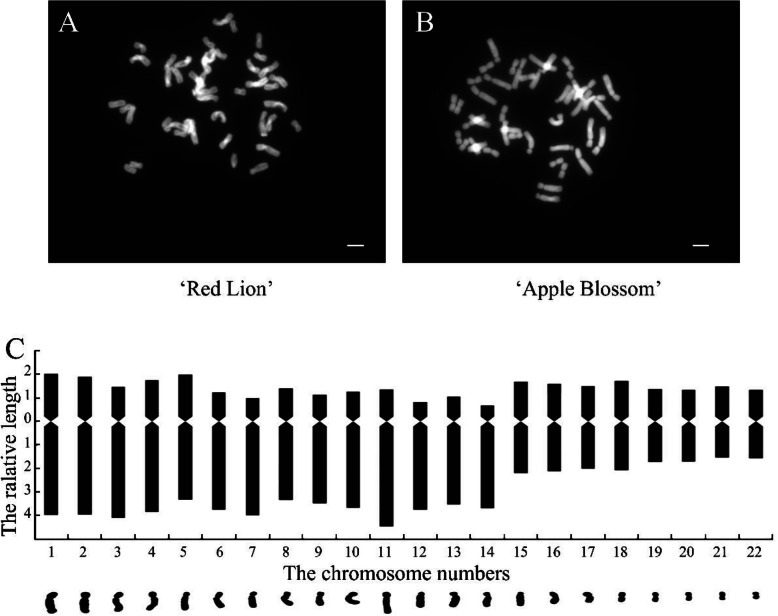
Microsome of ‘Red Lion’ nuclear chromosomes **a**, micrograph of ‘Apple Blossom’ nuclear chromosomes **b**, and karyotype pattern of the chromosomes in *Hippeastrum rutilum* c

The number of nuclear chromosomes of *Hippeastrum rutilum* in the two cultivars was observed under a microscope, and the chromosome type of both cultivars was tetraploid. The number of chromosomes in the nucleus was 44. The karyotype formula of the two cultivars was 2n = 4x = 44 = 18 m + 12sm + 14st (Fig. 1, Table 1). The average length of the chromosomes was between 2.902 and 5.988 μm, and the arm ratio was between 1.043 and 5.510. The ratio of the longest chromosome to the shortest chromosome was 2.06, and there were 6 pairs of chromosome arms with a ratio greater than 2:1, accounting for 55.5% of the total chromosomes. According to the karyotype analysis standard of Stebbins, it could be determined that the karyotype of *Hippeastrum rutilum* was 3B, and the karyotype asymmetry coefficient was 68.51%.

**Table 1:** The chromosome parameters of *Hippeastrum rutilum*

Numbers	Absolute length of chromosome (μm)			Relative length of chromosome (%)			Genomic length (μm)	Arm ratio	Centrome reposition
	Total length	Long arm	Short arm	Total length	Long arm	Short arm			
1	8.580	5.694	2.886	5.988	3.974	2.014	143.269	1.973	sm
2	8.387	5.679	2.708	5.854	3.964	1.890	143.269	2.097	sm
3	8.263	6.071	2.192	5.767	4.237	1.530	143.269	2.854	sm
4	8.006	5.509	2.497	5.588	3.845	1.743	143.269	2.206	sm
5	7.904	4.918	2.986	5.517	3.433	2.084	143.269	1.647	m
6	7.541	5.686	1.855	5.264	3.969	1.295	143.269	3.065	st
7	7.440	5.853	1.587	5.193	4.085	1.108	143.269	3.688	st
8	7.032	4.901	2.131	4.908	3.421	1.487	143.269	2.300	sm
9	6.617	5.008	1.609	4.618	3.495	1.123	143.269	3.112	st
10	7.066	5.265	1.801	4.931	3.675	1.257	143.269	2.924	sm
11	8.332	6.405	1.927	5.815	4.470	1.345	143.269	3.323	st
12	6.556	5.394	1.162	4.576	3.765	0.811	143.269	4.643	st
13	6.569	5.062	1.507	4.585	3.533	1.052	143.269	3.358	st
14	6.240	5.281	0.959	4.355	3.686	0.669	143.269	5.510	st
15	5.566	3.165	2.401	3.885	2.209	1.676	143.269	1.318	m
16	5.394	2.990	2.404	3.767	2.087	1.678	143.269	1.244	m
17	5.034	2.888	2.146	3.514	2.016	1.498	143.269	1.346	m
18	5.435	2.987	2.448	3.794	2.085	1.709	143.269	1.220	m
19	4.455	2.488	1.967	3.109	1.736	1.373	143.269	1.265	m
20	4.361	2.451	1.910	3.044	1.710	1.333	143.269	1.283	m
21	4.333	2.213	2.120	3.024	1.544	1.480	143.269	1.043	m
22	4.158	2.250	1.908	2.902	1.570	1.332	143.269	1.179	m

### Comparison of leaf morphological differences between two cultivars of *Hippeastrum rutilum*

During the growth period, the leaf tip angles, leaf sag angles, and leaf widths of the two cultivars of *Hippeastrum rutilum* were measured using measurement tools. The results showed that ‘Apple Blossom’, with drooping leaves, presented significantly larger values than ‘Red Lion’, with upright leaves, for the leaf tip angle, leaf sag angle, and leaf width (*P* < 0.05, Table 2).

**Table 2:** Morphological indexes (mean ± SD) of the two cultivars of *Hippeastrum rutilum* (*P* < 0.05)

Morphological index	Mean ± SD	
	‘Red Lion’	‘Apple Blossom’
Leaf tip angle (°)	48.20 ± 3.60b	56.20 ± 3.30a
Leaf width (cm)	4.34 ± 0.30b	4.82 ± 0.21a
Leaf sag angle (°)	37.80 ± 0.80b	49.20 ± 1.20a

### Comparison of Stomatal distribution characteristics between two cultivars of *Hippeastrum rutilum*

**Fig. 2:**
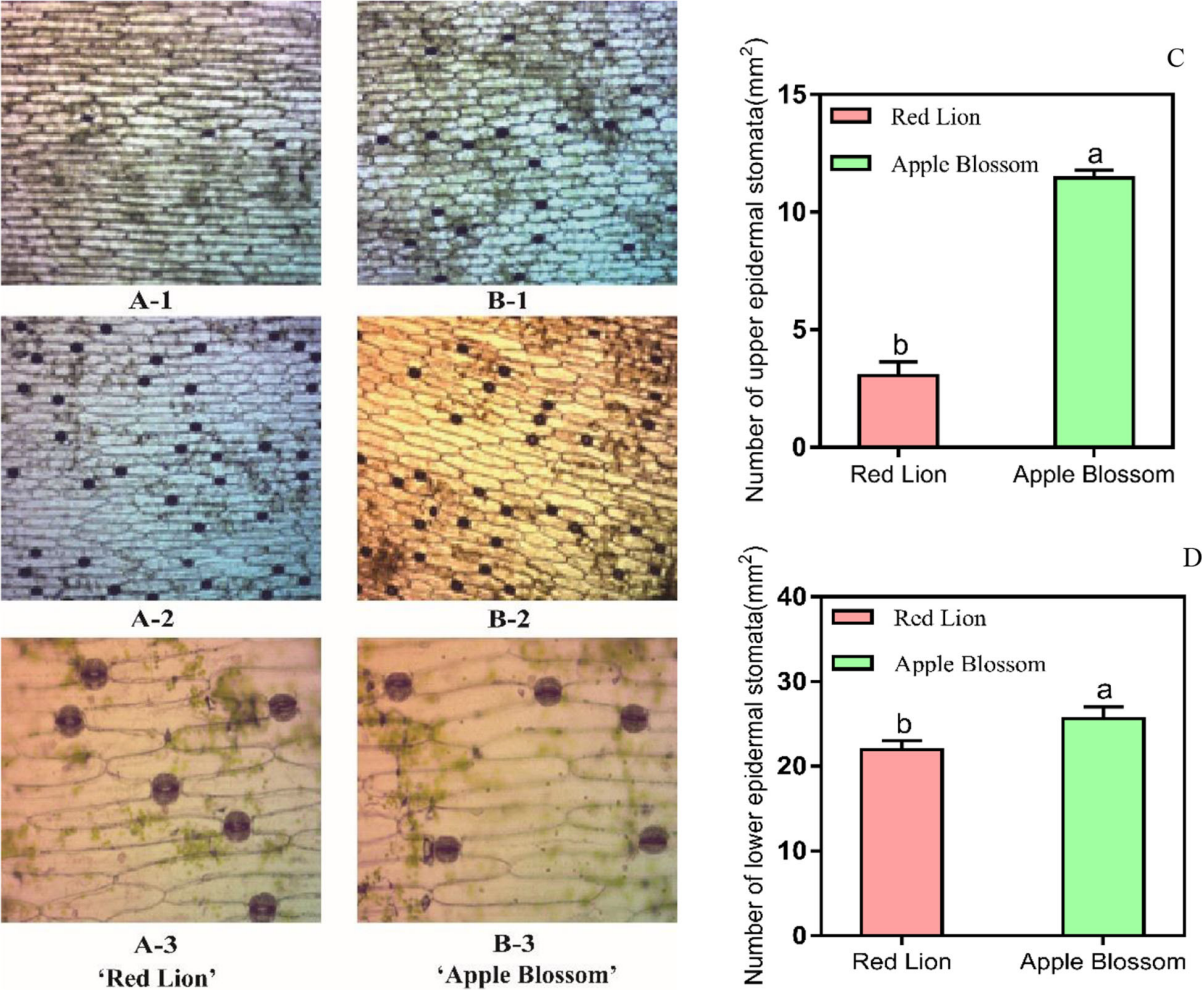
10X micrograph of the upper epidermal stomata of ‘Red Lion’ (**a**-1), 10X micrograph of the upper epidermal stomata of ‘Apple Blossom’ (**b**-1). 10X micrograph of the lower epidermal stomata of ‘Red Lion’ (**a**-2), 10X micrograph of the lower epidermal stomata of ‘Apple Blossom’ (**b**-2), 20X micrograph of the upper epidermal stomata of ‘Red Lion’ (**a**-3), 20X micrograph of the upper epidermal stomata of ‘Apple Blossom’ (**b**-3). Number of upper epidermal stomata per unit area in the two cultivars (*P* < 0.05, **c**, number of lower epidermal stomata per unit area in the two cultivars (*P* < 0.05, **d**. Total number of replicates used for this experiment was four

According to the obtained micrographs, the stomata of the epidermal cells of the two cultivars of *Hippeastrum rutilum* are composed of two half-moon-shaped guard cells, and there are no auxiliary guard cells (Fig. 2 b). A, B). The average stomatal density in the upper epidermal cells of ‘Red Lion’ was calculated to be 3.06, and the average stomatal density in the lower epidermal cells was 21.99. The average stomatal density in the upper epidermal cells of ‘Apple Blossom’ was 11.45, and the average stomatal density in the lower epidermal cells was 25.62. The average stomatal density in ‘Red Lion’ epidermal cells was significantly lower than that in ‘Apple Blossom’ (*P* < 0.05, Fig. 2 c, d). The average length of the stomata in the ‘Red Lion’ epidermis was 54.79 μm, and the average width was 45.81 μm. The average length of the stomata in ‘Apple Blossom’ was 58.92 μm, and the average width was 48.62 μm. The aspect ratios of the stomata of the two cultivars were very low, ranging from 1.09 to 1.44.

### Comparison of leaf cross-section structural characteristics between two cultivars of *Hippeastrum rutilum*

**Fig. 3:**
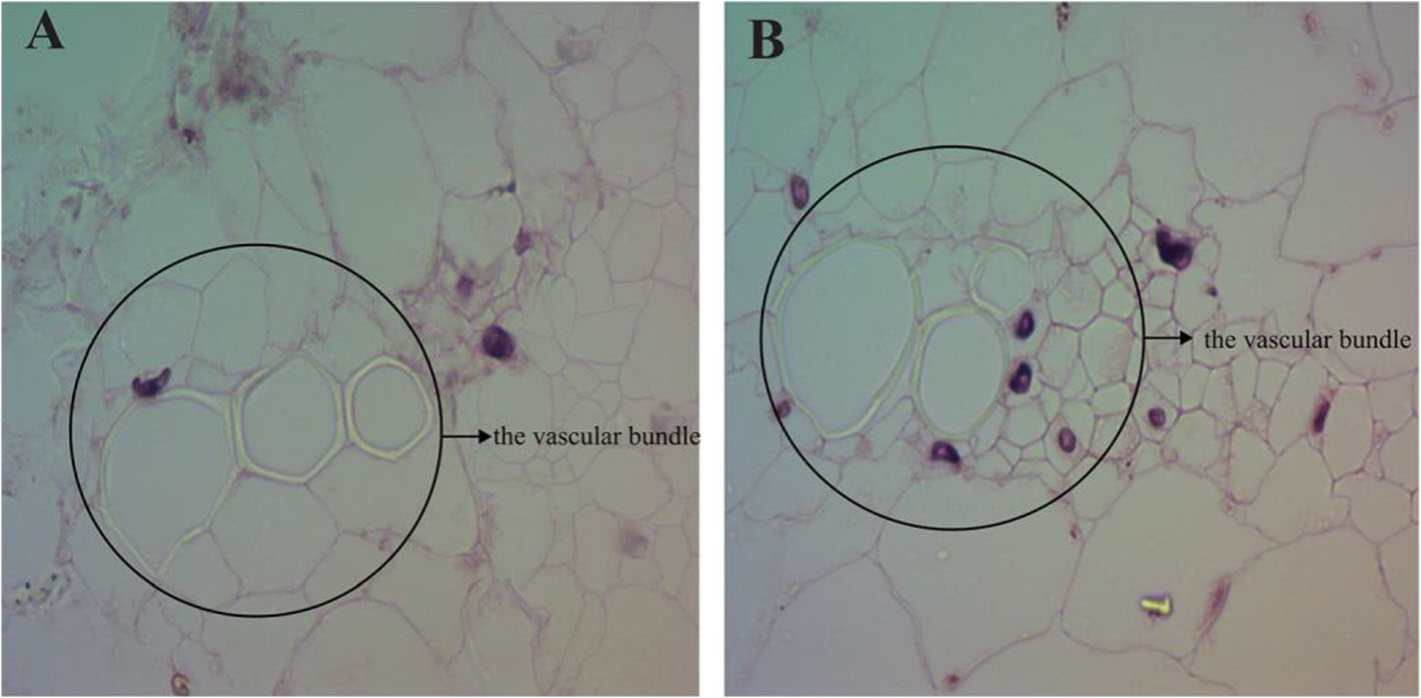
Cross-sectional structures of ‘Red Lion’ and ‘Apple Blossom’ leaves in the vascular bundle

The morphology of the vascular bundle cross-sections of the leaves of the two cultivars was observed under an optical microscope. The vascular bundle cell wall of ‘Red Lion’ was approximately hexagonal, and the cell wall of ‘Apple Blossom’ was nearly round. Compared with Red Lion’, the vascular bundle cells of ‘Red Lion’ were more regular and uniform in size. The cells surrounding the vascular bundles of ‘Red Lion’ were larger and more uniform than those surrounding the vascular bundles of ‘Apple Blossom’ (Fig. 3).

### Comparison of leaf relative water content and leaf lignin content between the two cultivars of *Hippeastrum rutilum*

**Fig. 4:**
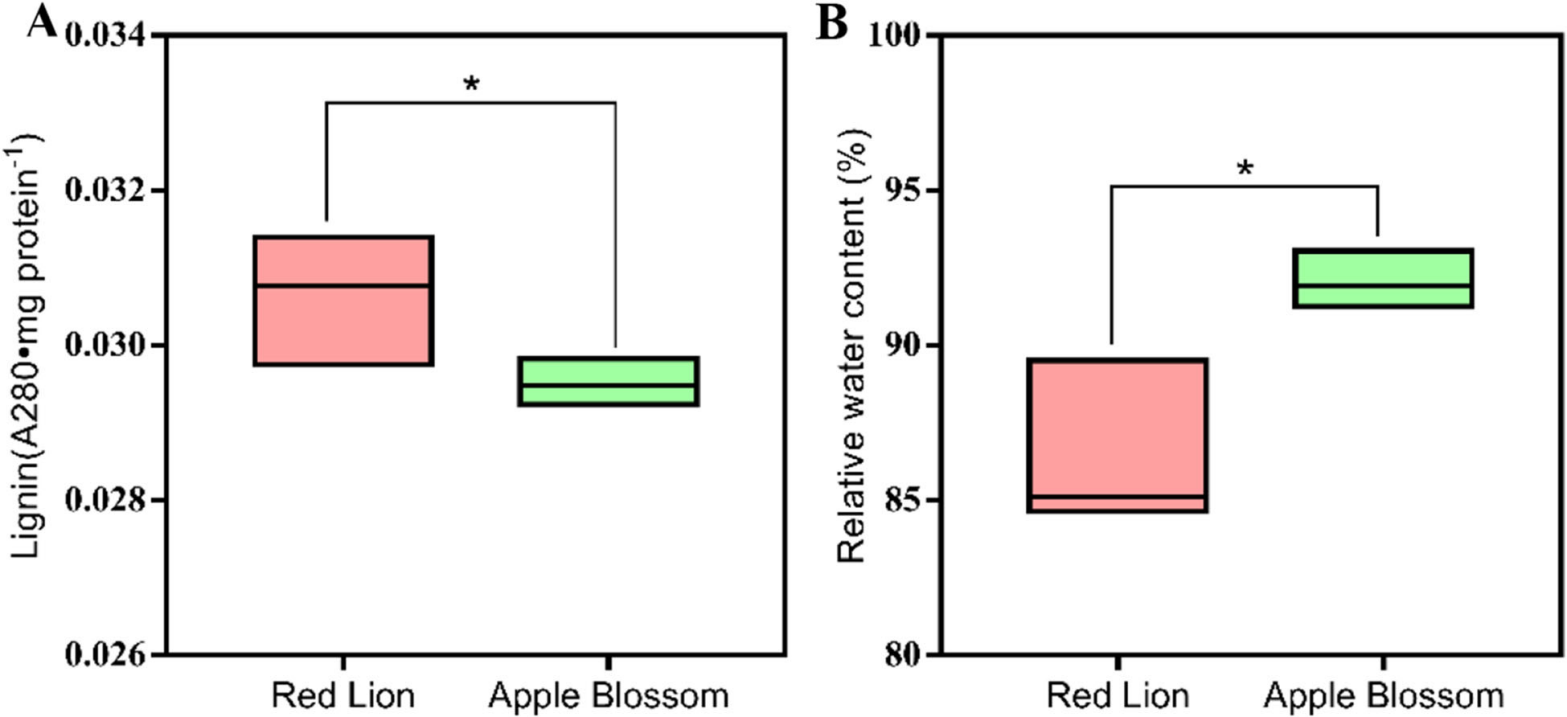
Lignin contents in the leaves of the two cultivars (*P* < 0.05, **a**, relative water contents in the leaves of the two cultivars (*P* < 0.05, **b**. Total number of replicates used for this experiment was four

The lignin content in the leaves of ‘Red Lion’, with upright leaves, was significantly higher than that of ‘Apple Blossom’, with leaf lodging (*P* < 0.05, Fig. 4 b). A). However, the leaf relative water content (RWC) of ‘Red Lion’, with upright leaves, was significantly lower than that of ‘Apple Blossom’, with drooping leaves (*P* < 0.05, Fig. 4 b). B).

### Comparison of chlorophyll content between the two cultivars of *Hippeastrum rutilum*

The Chl *a*, Chl *b* and Car contents of ‘Red Lion’ leaves were significantly higher than those of ‘Apple Blossom’ leaves (*P* < 0.05, Table 3). The ratio of the total Chl content in ‘Red Lion’ leaves to the total Chl content in ‘Apple Blossom’ leaves was approximately 2:1. The ratio of Chl a and Chl b in ‘Red Lion’ leaves was approximately 3:2, and the ratio of Chl a and Chl b in ‘Apple Blossom’ leaves was approximately 3:1.

**Table 3:** Chlorophyll content (Mean ± SD) in the leaves of the two cultivars (*P* < 0.05)

Parameters	Mean ± SD	
	Red Lion	Apple Blossom
Chl *a* [mg·g^−1^(FM)]	2.65 ± 0.06a	1.65 ± 0.22b
Chl *b* [mg·g^−1^(FM)]	1.78 ± 0.20a	0.49 ± 0.07b
Car [mg·g^−1^(FM)]	0.42 ± 0.02a	0.35 ± 0.05b
Chl(*a/b*) [mg·g^−1^(FM)]	1.50 ± 0.14b	3.37 ± 0.06a
Total Chl [mg·g^−1^(FM)]	4.87 ± 0.09a	2.15 ± 0.29b

*Car* carotenoids, *Chl* chlorophyll

### Comparison of chlorophyll fluorescence between the two cultivars of *Hippeastrum rutilum*

**Fig. 5:**
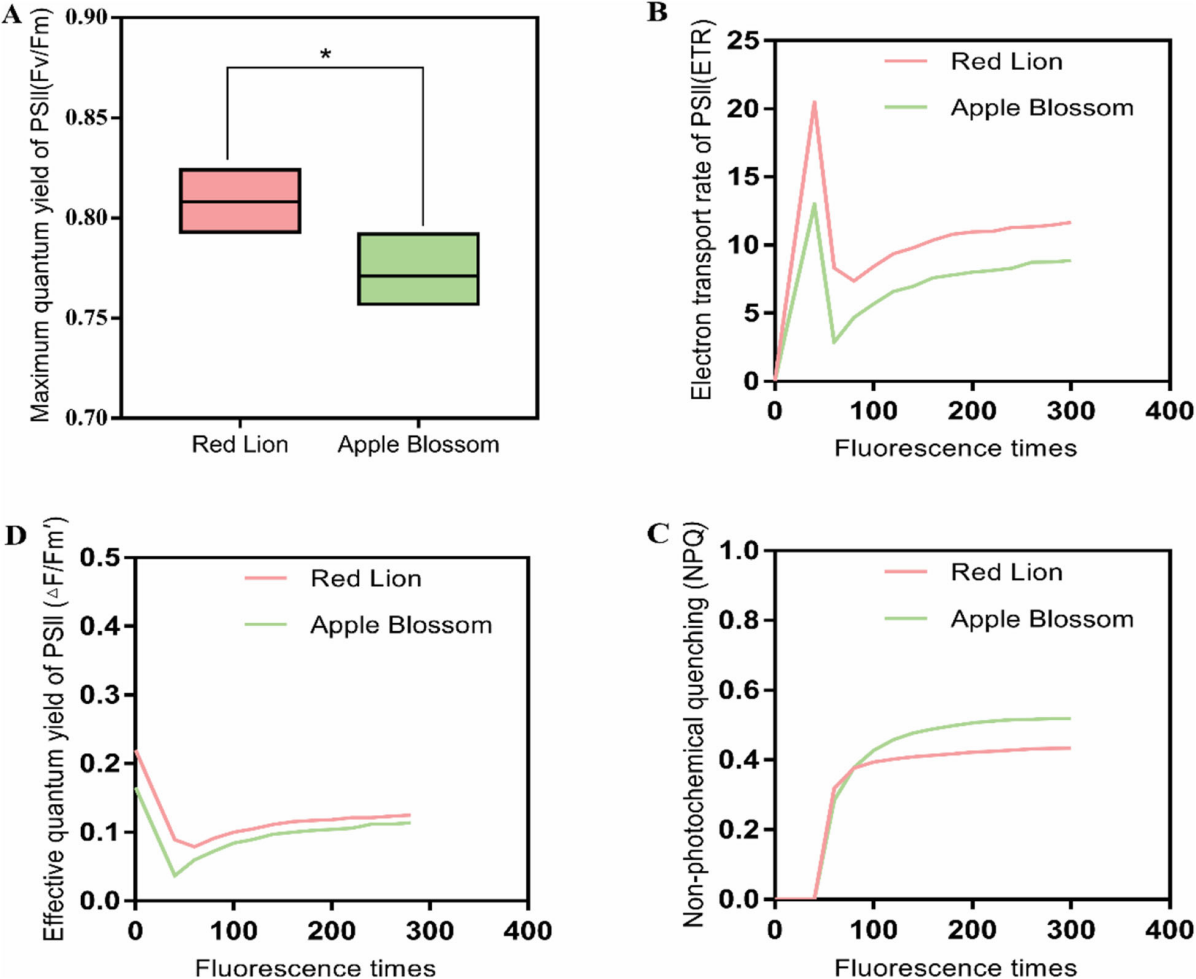
The maximum quantum yield of photosystem II photochemistry (Fv/Fm) in the two cultivars (*P* < 0.05, **a**, changes in the relative electron transfer rate (ETR) of the two cultivars **b**, changes in the nonphotochemical quenching (NPQ) of the two cultivars **c**, changes in the effective quantum yield of the photosystem II photochemistry (△−F/Fm′) of the two cultivars **d**. Total number of replicates used for this experiment was four

The chlorophyll fluorescence parameters of the two cultivars with different phenotypes were compared. The maximum quantum yield of the photosystem II photochemistry (Fv/Fm) of ‘Red Lion’, with upright leaves, was significantly greater than that of ‘Apple Blossom’, with leaf lodging (*P* < 0.05, Fig. 5 a). The value of Fv/Fm for ‘Red Lion’ was between 0.80–0.84, and the value for ‘Apple Blossom’ was below 0.80. The effective quantum yield of photosystem II photochemistry (△F/Fm′) and relative electron transport rate (ETR) parameters, which are positively related to the photosynthetic capacity of a plant, showed greater values in ‘Red Lion’ than in ‘Apple Blossom’ (*P* < 0.05, Fig. 5 b, D). However, nonphotochemical quenching (NPQ) parameters, which present a negative correlation with the plant photosynthetic capacity, showed greater values in ‘Apple Blossom’ than in ‘Red Lion’ (*P* < 0.05, Fig. 5 c). From the results for these parameters, it can be concluded that the photosynthetic capacity of ‘Red Lion’, with upright leaves, is greater than that of ‘Apple Blossom’, with leaf lodging.

## Discussion

*Hippeastrum rutilum* has a complex genetic background and exhibits diverse karyotypes, including diploid, triploid and tetraploid types [35, 36]. Phenotypic effects caused by karyotypic changes have been reported in plants such as Chinese large-flowered chrysanthemum (*Chrysanthemum* × *morifolium Ramat*.), *Arabidopsis thaliana*, and potato (*Solanum tuberosum*) plants regenerated from protoplasts, *Siraitia grosvenorii*, and *Hypericum perforatum* [37–41]. In the present study, we first performed a karyotype analysis of two cultivars with significantly different phenotypes, and both cultivars were found to be tetraploid, with no difference in their karyotype. It is speculated that the difference in the leaf lodging and upright traits between the two cultivars may not be due to a difference in their karyotypes. Plant lodging is related to the effects of many factors [42–44]. Although an influence of karyotypic changes on plant leaf lodging phenotype differences has been ruled out, plant leaf lodging resistance may be related to certain genes on a single chromosome, which needs further study [36, 45]. However, this difference may also be caused by other factors [44, 46]. We further observed and compared the leaf morphology and anatomical structure of the two cultivars. It was found that the values of morphological indicators such as the leaf tip angle, leaf width, and leaf sag angle in ‘Red Lion’, with upright leaves, were significantly smaller than those in ‘Apple Blossom’, with leaf lodging. The morphological characteristics of the leaves are closely related to the lodging characteristics of leaves [47, 48]. The anatomical structure of midvein vascular bundles was compared in the two cultivars. The vascular bundle structure of ‘Red Lion’ was found to be more uniform than that of ‘Apple Blossom’. The vascular bundle cells exhibited a uniform size, a regular cell wall shape, and denser surrounding cells. Compared with the vascular bundle cells of ‘Apple Blossom’, vascular bundle cells of ‘Red Lion’ had thicker walls and smaller vascular bundle voids in their center. These characteristics indicate that the carbohydrate content of the vascular bundle cell wall of ‘Red Lion’ is high, which may indicate that the stability of the vascular bundles of ‘Red Lion’ is greater. There is an important connection between leaf sagging and vascular bundle structure [49–52]. We can conclude from the differences in leaf morphology and anatomical structure that the upright and lodging traits of the two cultivars are closely related to leaf morphological characteristics. The ‘Red Lion’ leaves are narrower than those of ‘Apple Blossom’, and the angle of the ‘Red Lion’ leaf tip was smaller than that of the ‘Apple Blossom’ leaf tip. At the same time, by comparing the relative water content of the leaves of the two cultivars, it was found that the relative water content of the leaves of ‘Red Lion’ was significantly lower than that of ‘Apple Blossom’. The relative water content of plant tissues reflects the water status of the plant tissues and the water-holding capacity of the plants and is an important basis of plant drought resistance [53, 54]. Drought can promote an increase in carbohydrate contents in plant tissues to cope with drought stress. Carbohydrates are an important component of cell walls and vascular bundles, increasing the ability of plant leaves maintain their structure. The relative water content in the leaves increases the burden on the main vein to withstand gravity [55]. The relative water contents of plant tissues, the structure of the vascular bundles, the width of the leaves and the angle of the leaves all indicated that the “Red Lion” variety has some ability to withstand drought. These factors may indicate that the drought resistance of ‘Red Lion’ leaves is greater than that of ‘Apple Blossom’ leaves. These physiological morphological characteristics of the leaves may represent a strategy whereby ‘Red Lion’ copes with drought [56, 57]. The sag angle of ‘Red Lion’ leaves was smaller than that of ‘Apple Blossom’ leaves. By combining the lignin contents in the leaves of the two cultivars with the horizontal planing structure of the leaf vascular bundles, it was shown that ‘Red Lion’ leaves presented a greater hardening ability and higher carbohydrate content than ‘Apple Blossom’ leaves. Therefore, the comprehensive analysis of the internal and external morphological characteristics and physiological indicators of the plants showed that the difference between these factors in ‘Red Lion’ and ‘Apple Blossom’ is closely related to lodging resistance. Many studies have confirmed that the content of lignin is an important physiological indicator of plant lodging resistance [58–61]. We compared the lignin contents of the leaves of two cultivars with different phenotypes and found that the lignin content of ‘Red Lion’, with upright leaves, was significantly higher than that of ‘Apple Blossom’, with leaf lodging. This provides a strong basis for exploring the mechanisms underlying the differences between the different phenotypes of the cultivars. Comprehensive analyses showed that compared to ‘Red Lion’, with upright leaves, ‘Apple Blossom’, with leaf lodging, exhibits a greater leaf width, greater leaf sag angle, and higher relative water content (RWC), while its vascular bundle cell structure shows low stability, and the content of lignin is low. These factors may weaken the anti-lodging ability of the leaves, thereby increasing the probability of ‘Apple Blossom’ lodging [3, 62, 63]. Lodging can have serious effects on plant yield and quality [64, 65]. Photosynthesis is an important pathway for the accumulation of organic matter in plants. Does lodging affect the ability of plants to photosynthesize, in turn affecting plant quality and yield? We determined the photosynthesis-related indicators of chlorophyll content, leaf stomatal density, and chlorophyll fluorescence parameters in the two cultivars. The chlorophyll content, Fv/Fm and other indicators showed that photosynthesis was higher in ‘Red Lion’ than in ‘Apple Blossom’. However, the observation of the density of the stomata in the leaves of the two cultivars showed that the stomatal density of ‘Apple Blossom’ was greater than that of ‘Red Lion’. Plant stomatal density is inextricably linked to plant respiration and transpiration [66, 67], and the results therefore indicate that it is possible that ‘Apple Blossom’ exhibits increased respiration compared with ‘Red Lion’. Therefore, it is very likely that the accumulation of organic matter is reduced due to increased respiration and reduced photosynthesis, and the accumulation of organic matter in plants further affects their yield and quality [68]. Studies have shown that the germination rate of ‘Apple Blossom’ seeds is significantly lower than that of ‘Red Lion’ seeds, which may also be related to leaf lodging.

## Conclusion

Our results show that there are significant differences in morphological structure, physiological characteristics, and photosynthesis between two cultivars of *Hippeastrum rutilum* with different phenotypes. ‘Red Lion’, with upright leaves, exhibits a higher lignin content, and ‘Apple Blossom’, with sagging leaves, exhibits a lower lignin content. The cultivar with upright leaves shows a low sag angle in its morphological structure, a more regular vascular bundle structure and thicker cell walls. Cultivars that readily show leaf lodging exhibit large sag angles, irregular vascular bundle structures, and thin cell walls. The stomatal structure, chlorophyll content, and chlorophyll fluorescence parameters of the leaves of the two cultivars were compared. It was found that the photosynthesis in ‘Apple Blossom’, affected by lodging, was significantly increased compared with than that of ‘Red Lion’, and lodging affected the photosynthetic capacity of ‘Apple Blossom’. Such work may provide empirical and theoretical support for artificial cultivation and variety improvement in *Hippeastrum rutilum*.

## Data Availability

The datasets used and/or analysed during the current study available from the corresponding author on reasonable request.
